# Elastic Boundary Control in Acoustic Waveguides for High-Fidelity Physical-Layer Telemetry in Downhole Sensor Networks

**DOI:** 10.3390/s26123826

**Published:** 2026-06-16

**Authors:** Hao Geng, Yingjian Xie, Zhihao Wang, Hu Han, Dong Yang

**Affiliations:** 1 Hubei Key Laboratory of Oil and Gas Drilling and Production Engineering, Yangtze University, Wuhan 430100, China; 2022710259@yangtzeu.edu.cn (H.G.); 2024720459@yangtzeu.edu.cn (Y.X.); 2025720454@yangtzeu.edu.cn (Z.W.); hanhu@yangtzeu.edu.cn (H.H.); 2 School of Petroleum Engineering, Yangtze University, Wuhan 430100, China; 3 State Key Laboratory of Low Carbon Catalysis and Carbon Dioxide Utilization, Yangtze University, Wuhan 430100, China

**Keywords:** coiled tubing, acoustic telemetry, downhole sensor networks, MWD, telemetry bit error rate, contact nonlinearity, elastic preload

## Abstract

**Highlights:**

**What are the main findings?**

- A gravity-independent elastic boundary control method was proposed for downhole sensor networks to suppress physical-layer waveform clipping in horizontal wells.
- Full-scale circulation testing demonstrated that the optimized physical baseline reduced waveform asymmetry to 2.1% and total harmonic distortion to 1.8%.
- Shifting the structural natural frequency to 2850 Hz achieved a 12.0 dB SNR improvement by physically isolating the system from low-frequency pump interference.

**What are the implications of the main findings?**

- The resulting 12.0 dB physical-layer gain significantly expands theoretical channel capacity, elevating data throughput limits for high-frequency geosteering sensor payloads.
- Enhancing source-signal phase fidelity suppresses carrier phase distortion, theoretically reducing the telemetry bit error rate of MWD networks by several orders of magnitude.
- Eliminating heavy tungsten blocks and minimizing harmonic crosstalk guarantee seamless structural and frequency domain compatibility with heterogeneous sensor arrays in complex tool strings.

**Abstract:**

In the development of deep shale gas horizontal wells, precise geo-steering relies heavily on downhole sensor networks to acquire extensive formation and engineering parameters. Coiled tubing (CT) provides a promising acoustic waveguide for downhole sensing systems, but conventional acoustic sources rely on gravity-induced interfacial preload. Under highly deviated or horizontal well conditions, the loss of the axial gravity component may induce contact–nonlinearity instability, resulting in waveform distortion and spectral pollution. To address this limitation, a constant-stiffness preloading method based on elastic compliance control is proposed, together with a modal reconstruction strategy achieved by removing high-density tungsten blocks. A fluid–solid coupled dynamic model incorporating contact nonlinearity is established to reveal the dynamic separation mechanism of the acoustic source interface under varying gravity-vector conditions. Wave spring assemblies are then used to reconstruct the mechanical boundary and physically suppress time-domain clipping. Full-scale ground circulation experiments on a 1500 ft CT string show that the proposed method decouples acoustic-source performance from wellbore trajectory. Waveform asymmetry is reduced from 18.4% to 2.1%, and total harmonic distortion decreases from 12.5% to 1.8%. In addition, the first-order longitudinal natural frequency is shifted from 420 Hz to 2850 Hz, avoiding low-frequency pump noise and achieving a 12 dB SNR improvement. This physical-layer gain provides an optimized signal baseline for receiver-end demodulation algorithms. Ultimately, this study provides a robust physical-layer solution for acoustic telemetry in complex deep-earth environments, advancing the reliability of data interaction in downhole sensing systems.

## 1. Introduction

With global energy exploration moving toward deep, ultra-deep, and unconventional reservoirs, real-time downhole-to-surface data transmission has become a fundamental requirement for geosteering and operational condition monitoring in deep horizontal wells [1]. In the development of deep shale gas and tight gas reservoirs, long horizontal sections are not only the main zones for productivity enhancement but also information-blind intervals for geosteering and engineering risk control [2]. In such confined downhole environments, establishing a high-speed and reliable data transmission link from the bottomhole to the surface is essential for acquiring formation lithology parameters, monitoring hydraulic fracture propagation, and evaluating wellbore stability [3]. In the architecture of modern Measurement While Drilling (MWD) and sensing systems, this transmission link essentially constitutes an integrated downhole information sensing network. The complete network topology includes five core functional nodes. First, downhole multi-source sensor arrays acquire raw formation lithology and engineering dynamic parameters in real time. The MWD main control unit then processes these signals digitally and performs carrier modulation. Next, the physical-layer transducer converts electrical energy into acoustic energy and excites elastic waves. These waves propagate to the surface through the coiled tubing waveguide. Finally, a surface probe captures the signals at high sampling rates and demodulates them to complete data transmission. With the increasing automation of coiled tubing operations, the demand for high-frequency, real-time, bidirectional telemetry has become increasingly urgent [4].

Among various downhole wireless transmission technologies, acoustic telemetry has attracted considerable attention because of its unique physical characteristics. Unlike mud pulse telemetry (MPT), which relies on a continuous fluid column, acoustic telemetry (AT) uses the metallic tubular string itself as an elastic waveguide [5,6]. Under gas-dominated operating conditions, such as underbalanced drilling, foam-fluid sand cleanout, and high gas–liquid ratio production logging, MPT may fail because of the absence of a continuous liquid column, whereas AT offers irreplaceable transmission advantages [7,8]. In addition, compared with electromagnetic telemetry, which suffers from exponential attenuation with well depth and is easily shielded by formation resistivity, acoustic waves exhibit relatively low attenuation in metallic waveguides, making them a promising carrier for data transmission in deep and ultra-deep wells [9,10].

The theoretical foundation of this acoustic transmission relies on the fundamental physics of guided-wave acoustics and piezoelectric electromechanical coupling. Classical elastodynamic theories establish that cylindrical waveguides support highly dispersive and multimodal acoustic propagation, dictating the baseline channel characteristics [11]. Concurrently, the generation of these elastic waves fundamentally depends on the transient dynamics of piezoelectric transducers operating under varying structural constraints [12,13]. While recent application-oriented studies have extensively explored signal modulation and downhole network implementations, a critical physical-layer gap remains regarding the dynamic boundary stability of the acoustic source itself.

However, within the aforementioned downhole sensing network, the mechanical coupling interface between the acoustic transmitter and the waveguide wall represents a critical physical-layer node governing energy transduction. Existing studies on acoustic transmission mechanisms are mostly based on vertical-well assumptions and generally neglect the influence of wellbore trajectory variation on the dynamic boundary conditions of the acoustic source [14,15]. Conventional piezoelectric transmitters commonly employ high-mass tungsten alloy blocks as reaction elements, using their gravity component in the vertical direction to provide static preload and maintain mechanical closure at the transducer interface [16]. When the operating environment extends to horizontal or extended-reach wells, the gravity vector becomes nearly perpendicular to the axial direction of the tubular string, causing the effective axial preload component to approach zero [17]. This abrupt change in boundary conditions induces nonlinear dynamic instability at the contact interface [18]. From a sensor-system perspective, the resulting periodic interfacial separation and time-domain waveform clipping do not merely represent localized mechanical distortion; instead, they induce severe physical-layer source degradation across the entire telemetry link. The transmission carrier suffers severe spectral pollution at the point of generation. This means backend digital filtering and adaptive demodulation algorithms lose their underlying signal fidelity [19,20]. Consequently, this physical-layer source degradation constitutes a primary bottleneck restricting the operational reliability of the entire downhole sensing network [21].

Furthermore, the low-frequency modal characteristics caused by conventional tungsten-containing structures further degrade the signal-to-noise ratio (SNR) [22]. The high-density tungsten block lowers the first-order longitudinal natural frequency of the system, making it highly susceptible to resonant coupling with strong low-frequency fluid noise generated by drilling pumps [23]. Current research has mainly focused on back-end signal-processing methods, including orthogonal frequency-division multiplexing modulation, machine-learning- or deep-learning-based adaptive recognition and denoising algorithms, and various optimization search strategies [24]. Although digital signal processing can partially mitigate noise interference, it cannot eliminate the physical-layer degradation of the source signal caused by contact nonlinearity at its origin [25].

To address these challenges, rather than merely implementing localized mechanical modifications, this study focuses on the structural reconstruction of the mechanical boundary at the transducer interface, which represents the most vulnerable physical-layer node within the downhole information sensing network. Specifically, a gravity-independent constant-stiffness preloading and modal reconstruction strategy is proposed for coiled tubing acoustic telemetry. This approach aims to establish a highly linear physical-layer fundamental link directly at the acoustic source, thereby guaranteeing reliable real-time data interaction for massive downhole sensor payloads under complex wellbore trajectories. The effectiveness of the proposed method is validated through comprehensive modeling and full-scale ground circulation testing. The primary contributions of this work are summarized as follows:

(1) A dynamic model of the transmission interface considering contact nonlinearity is established to explain the formation mechanism of preload failure and spectral pollution under deep horizontal well conditions.
(2) A gravity-independent preloading method based on wave springs is proposed, in which a constant-stiffness boundary is used to replace the conventional gravity-dependent preload boundary.
(3) A tungsten-free lightweight modal reconstruction strategy is developed to shift the system natural frequency toward a higher-frequency range, thereby reducing coupling with low-frequency pump-induced noise.
(4) The effectiveness of the proposed method is validated using a 1500 ft full-scale experimental platform. The optimized structure shows clear advantages in waveform symmetry, harmonic suppression, and SNR improvement.

## 2. Materials and Methods

This section first establishes a fluid–solid coupled dynamic model of the CT acoustic transmission interface incorporating contact nonlinearity, with the aim of revealing the underlying physical mechanism by which gravity-dependent preload failure induces spectral pollution under complex wellbore trajectories. Subsequently, a boundary control strategy based on elastic compliance and a physical modal reconstruction method are proposed. The configuration of the ground circulation experimental platform used for full-link performance validation is also described in detail.

### 2.1. Contact–Nonlinear Dynamics of the Acoustic Transmission Interface Under Varying Orientations

This study employs a piezoelectric ceramic stack as the primary power source for acoustic excitation. According to linear piezoelectric theory, the conversion of electrical energy into mechanical energy can be constitutively described by the second type of piezoelectric equation under the longitudinal vibration mode [26]:

(1) $$S_{3}=s_{33}^{E}T_{3}+d_{33}E_{3},$$

where $S_{3}$ is the axial strain along the polarization axis, dimensionless; $s_{33}^{E}$ is the elastic compliance coefficient under a constant electric field, m^2^/N; $T_{3}$ is the axial mechanical stress, Pa; $d_{33}$ is the piezoelectric strain constant, C/N; and $E_{3}$ is the driving electric field intensity, V/m.

**Figure 1:**
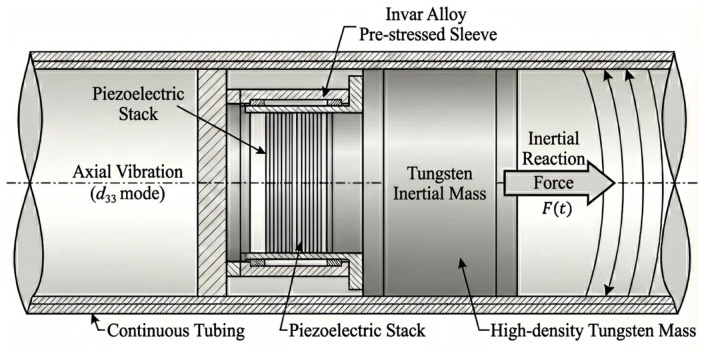
Cross-sectional structure of the conventional gravity-dependent piezoelectric acoustic transmitter.

The piezoelectric stack is composed of 20 PZT-5H ceramic plates, each measuring 15 mm × 15 mm × 2 mm, arranged in a mechanical series and electrical parallel configuration. This layout provides an optimal balance of axial force and displacement amplitude for exciting longitudinal elastic waves within the coiled tubing waveguide. PZT-5H was selected as the active material for its high piezoelectric strain constant, mechanical robustness, and a Curie temperature exceeding 190 °C, ensuring operational stability under typical downhole vibration and temperature conditions. While alternative materials such as PMN-PT single crystals offer higher piezoelectric moduli, they exhibit lower Curie temperatures and increased brittleness, which significantly reduce their reliability in harsh downhole environments and render them unsuitable for this application. The internal structure of the acoustic transmitter, as illustrated in Figure 1, comprises the piezoelectric stack, an Invar alloy pre-stressed sleeve, and a tungsten inertial reaction mass. The pre-stressed sleeve applies a constant compressive load to the piezoelectric stack to prevent tensile failure during high-amplitude vibration.

Assuming that the piezoelectric stack consists of N ceramic plates mechanically connected in series, when a time-varying driving voltage U(t) is applied, the maximum dynamic blocking force $F_{block}(t)$ generated under ideal rigid constraints serves as the primary driving force source for radiating acoustic energy from the system into the external waveguide [27]:

(2) $$F_{block}(t)=K_{stk}\cdot N\cdot d_{33}\cdot U(t),$$

where $F_{block}(t)$ is the blocking force, N; $K_{stk}$ is the equivalent axial stiffness of the piezoelectric stack, N/m; U(t) is the time-varying driving voltage, V.

However, in actual deep-well environments, the coupling state between the end cap of the acoustic transducer and the tubular wall directly governs the energy transfer efficiency. Based on contact mechanics, this impedance interface can essentially be regarded as a unilateral nonlinear constraint system regulated by the axial static preload. When the contact surface is equivalent to a non-ideal rough surface, the transient interfacial contact stiffness $K_{c}$ and the normal static preload $F_{pre}$ follow a Hertzian contact power–law relationship [28]:

(3) $$K_{c}=\alpha \cdot F_{pre}^{1/3},$$

where $K_{c}$ is the interfacial contact stiffness, N/m; $F_{pre}$ is the static axial preload acting on the interface, N; and α is the contact coefficient related to the geometric and physical properties of the contact materials, N^2/3^/m. This physical characteristic, in which the interfacial stiffness is highly dependent on the static preload, constitutes the most mechanically vulnerable link in the entire acoustic transmission chain. By treating the coiled tubing as a one-dimensional elastic waveguide, a lumped-parameter dynamic topology incorporating the coupling effect of the contact interface can be established.

**Figure 2:**
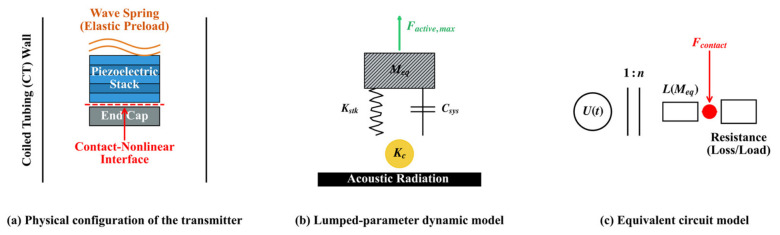
Physical structure and dynamic equivalent model of the acoustic transmission system.

To intuitively describe the physical components corresponding to the above dynamic equations and the electromechanical coupling path, an equivalent system model is established, as shown in Figure 2. Figure 2a shows the physical configuration of the transmitter incorporating the elastic preloading component, clearly indicating the physical interface where contact nonlinearity occurs. Figure 2b abstracts this configuration into a lumped-parameter dynamic model, in which the contact stiffness $K_{c}$ and the stack stiffness $K_{stk}$ are connected in series and jointly determine the force transmission efficiency of the system. Figure 2c further presents the corresponding Mason equivalent circuit model, which maps the mechanical impedance into electrical parameters and provides a theoretical basis for the subsequent frequency domain analysis.

As indicated by the dynamic topology in Figure 2b, the total restoring force of the system depends on the coupling effect between the piezoelectric stack stiffness $K_{stk}$ and the contact stiffness $K_{c}$. Under ideal linear conditions, both stiffness values remain constant. However, the contact–nonlinear interface indicated by the red dashed line in Figure 2a represents the only mechanically vulnerable link in the entire acoustic transmission chain. Once sufficient static preload is absent, $K_{c}$ undergoes a nonlinear jump with vibration displacement. This dynamic instability of the mechanical parameter is the physical origin of impedance mismatch in the equivalent circuit shown in Figure 2c, and further leads to the spectral pollution discussed in the following sections.

To isolate the primary mechanism of axial contact nonlinearity and ensure the tractability of the lumped-parameter dynamic model, several fundamental physical assumptions are explicitly established. First, the structural damping of the piezoelectric stack and the metallic components is neglected. This simplification allows the theoretical analysis to focus entirely on the macroscopic energy dissipation induced by interfacial separation rather than inherent material attenuation. Second, the coiled tubing waveguide is assumed to be a perfectly elastic continuum operating strictly within its linear proportional limit under the applied high-frequency acoustic excitation. Third, complex frictional nonlinearities, such as Coulomb friction between the tool housing and the borehole wall, are excluded from the current dynamic topology. While these structural assumptions effectively uncouple the core problem of gravity-induced preload failure, they present inherent limitations under extreme downhole conditions.

Conventional acoustic telemetry tools commonly rely on the gravity component of high-mass tungsten alloy blocks to provide static preload, thereby maintaining mechanical closure at the transducer interface. As the well inclination angle θ increases, the effective axial preload $F_{eff}(\theta )$ acting on the piezoelectric stack interface shows a significant decreasing trend [29]:

(4) $$F_{eff}(\theta )=F_{gravity}\cdot \cos\;\theta +F_{fric},$$

where $F_{eff}(\theta )$ is the effective axial preload, N; $F_{gravity}$ is the total gravitational force acting on the gravity-preloaded mass block, N; and $F_{fric}$ is the static friction force between the structural components of the system, N.

**Figure 3:**
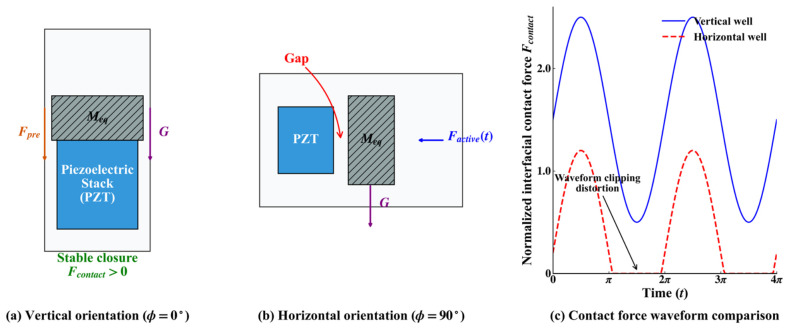
Schematic of contact nonlinearity and gravity-induced failure mechanism.

When the drilling trajectory extends into an extended-reach horizontal section, the gravity vector becomes perpendicular to the axial direction of the tubular string, causing the effective preload component to nearly vanish. Figure 3 illustrates the mechanism of contact nonlinearity and gravity-induced preload failure.

As shown in Figure 3a,b, during dynamic excitation, the instantaneous contact force $F_{contact}(t)$ at the interface becomes highly unbalanced:

(5) $$F_{contact}(t)=F_{pre}+F_{active,max}\cdot \sin(\omega t),$$

where $F_{contact}(t)$ is the instantaneous contact force, N; $F_{active,max}$ is the amplitude of the axial excitation force generated by the piezoelectric stack, N; ω is the angular frequency of the signal, rad/s; and t is time, s.

As shown in Figure 3c, once the dynamic inertial force during the negative half-cycle exceeds the weak residual preload, the instantaneous contact force at the interface drops to zero, resulting in microsecond-scale dynamic separation between the piezoelectric stack and the tool end cap. This physical separation forces the displacement response to exhibit flat-topped waveform distortion caused by clipping. According to Fourier series theory, asymmetric waveform clipping inevitably induces substantial energy spreading in the frequency domain. A considerable portion of the fundamental frequency energy is transferred to high-order harmonic bands, leading to a sharp increase in total harmonic distortion and eventually evolving into severe broadband spectral pollution.

**Figure 4:**
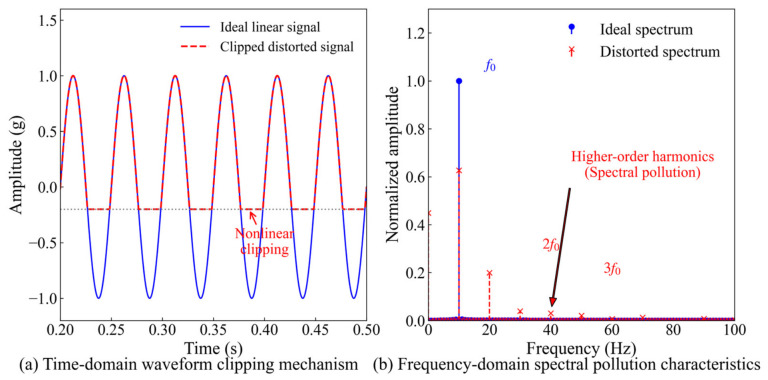
Schematic of waveform clipping and spectral pollution mechanism.

According to signal processing theory, nonlinear clipping of the time-domain waveform inevitably leads to energy spreading in the frequency domain. Figure 4 illustrates the mapping relationship between waveform clipping induced by contact nonlinearity and spectral pollution.

As shown in Figure 4a, the ideal linear signal maintains a continuous and symmetric sinusoidal waveform, whereas the distorted signal caused by contact nonlinearity exhibits obvious clipping during the negative half-cycle. This indicates that dynamic separation at the interface disrupts the continuity and amplitude symmetry of the time-domain response at the transmitter end. Figure 4b shows that the energy of the ideal spectrum is mainly concentrated at the fundamental frequency $f_{0}$. After clipping distortion occurs, however, the signal energy spreads toward ${2f}_{0}$, ${3f}_{0}$, and higher-order harmonic bands, exhibiting clear spectral pollution characteristics. This suggests that spectral pollution is essentially a redistribution of fundamental frequency energy caused by nonlinear instability of the contact boundary at the transmitter end. Therefore, receiver-end filtering or back-end signal processing alone cannot eliminate this type of distortion at its source. It is necessary to maintain stable interfacial contact through elastic boundary control and to suppress waveform clipping and harmonic spreading at the physical layer.

### 2.2. Elastic Boundary Control and Physical Modal Reconstruction Strategy

To eliminate the waveform-clipping pathway at its source, this study proposes a constant-stiffness boundary reconstruction method based on elastic compliance control. By incorporating a high-stiffness wave spring assembly into the acoustic excitation structure, an elastic-potential-energy-based preload $F_{pre}^{\prime }$ that is completely independent of the wellbore trajectory is provided for the system:

(6) $$F_{pre}^{\prime }=K_{spr}\cdot \Delta x_{ini},$$

where $F_{pre}^{\prime }$ is the optimized constant interfacial preload, N; $K_{spr}$ is the equivalent series stiffness of the wave spring assembly, N/m; and $\Delta x_{ini}$ is the initial compression displacement, m. The core of this stiffness-boundary design is to decouple the mechanical preload from the gravity vector. Through a dynamic compliance compensation mechanism, the inertial separation force is counteracted, allowing the system to operate within the linear contact regime throughout the entire excitation cycle.

**Figure 5:**
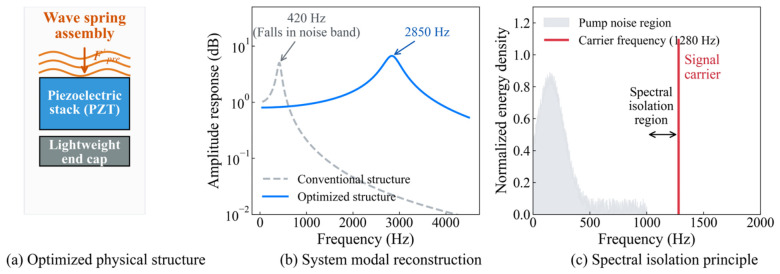
Optimized design and modal reconstruction diagram.

Furthermore, to address the susceptibility of conventional high-mass tungsten-containing structures to coupling with low-frequency fluid noise, this study implements a physical frequency avoidance strategy by minimizing the equivalent mass. Based on single-degree-of-freedom vibration theory, the first-order longitudinal natural frequency $\omega_{n}^{\prime }$ of the system is related to the equivalent mass $M_{eq}^{\prime }$ as follows [30]:

(7) $$\omega_{n}^{\prime }=\sqrt{\frac{K_{sys}}{M_{eq}^{\prime }}}\gg \omega_{n},$$

where $\omega_{n}^{\prime }$ is the first-order longitudinal natural frequency of the optimized system, Hz; $K_{sys}$ is the total equivalent longitudinal stiffness of the transmitter system, N/m; and $M_{eq}^{\prime }$ is the optimized equivalent vibrating mass, kg. By removing the high-density tungsten block and reconstructing the boundary constraint, the equivalent vibrating mass is substantially reduced while the overall dynamic stiffness is maintained, thereby shifting the natural frequency from 420 Hz to 2850 Hz. This upward migration of the natural frequency creates a broad physical isolation region between the high-noise fluid frequency band and the signal carrier, enabling inherent suppression of pump-induced noise. The principle is illustrated in Figure 5.

As shown in Figure 5a, the optimized acoustic transmission structure introduces a wave spring assembly above the piezoelectric stack and replaces the conventional high-mass tungsten block with a lightweight tail seat. Through pre-compression energy storage, the wave spring provides a continuous elastic preload to the contact interface between the piezoelectric stack and the end cap. This converts the interface closure mechanism from conventional gravity-vector control to elastic boundary control, thereby reducing the risk of dynamic separation caused by the degradation of effective axial preload under horizontal well conditions. Figure 5b further presents the difference in frequency response before and after system modal reconstruction. The first-order longitudinal natural frequency of the conventional tungsten-containing structure is located in the low-frequency region of approximately 420 Hz, where it can easily overlap with pump-induced fluid noise. In contrast, after removing the high-density tungsten block, adjusting the equivalent vibrating mass, and reconstructing the boundary constraint, the first-order longitudinal natural frequency of the optimized structure shifts to approximately 2850 Hz, clearly moving away from the low-frequency pump-induced noise-sensitive region. Figure 5c illustrates the physical frequency avoidance mechanism from the perspective of spectral distribution. Pump-induced noise energy is mainly concentrated in the low-frequency range, whereas the 1280 Hz communication carrier is located in a relatively quiet frequency region, forming a clear spectral isolation band between the two. Therefore, the proposed optimization scheme does not rely solely on back-end filtering algorithms for noise suppression. Instead, it improves source-signal fidelity and noise resistance in the acoustic telemetry link through two physical-layer pathways: stabilizing the contact interface using elastic preloading and reducing noise coupling through modal reconstruction.

### 2.3. Experimental Setup for Full-Link Acoustic Transmission Through Coiled Tubing

**Figure 6:**
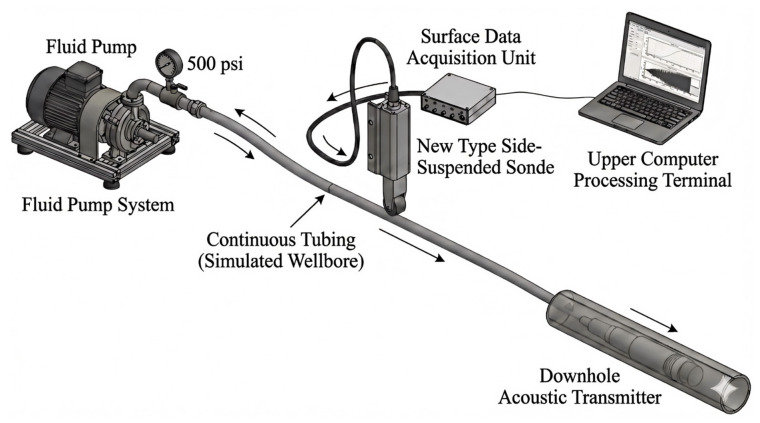
Full-scale system for coiled tubing acoustic telemetry.

To verify the effectiveness of the elastic preloading strategy in suppressing contact nonlinearity under horizontal well conditions, a full-scale CT acoustic transmission test system was constructed, as shown in Figure 6.

**Figure 7:**
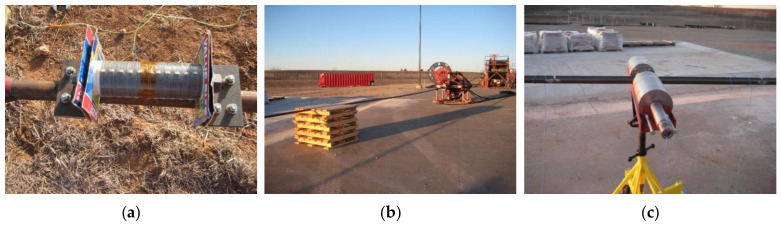
Key components of the full-scale experimental system. (**a**) Acoustic wave emission device. (**b**) Coiled tubing used in the experiment. (**c**) Acoustic receiving probe.

Figure 7 shows the three key components of the experimental system. The physical architecture strictly follows the transmission logic of the acoustic channel.

The physical architecture of the experimental system strictly follows the transmission logic of the acoustic channel. First, the acoustic transmitter shown in Figure 7a integrates a wave spring assembly. As the signal transmission carrier, Figure 7b shows a QT-900 coiled tubing waveguide (QT-900, Baoji Petroleum Steel Pipe Co., Ltd., Baoji, China) with a total length of 457 m. Fluid circulation inside the tubing was maintained by a triplex plunger pump at a flow rate of 0.5 bpm, thereby introducing realistic low-frequency, high-energy pump-induced interference noise. Signal acquisition was performed using the acoustic receiving probe shown in Figure 7c, which was installed at the end of the waveguide. Combined with a micro-lever amplification structure and a 50 kHz high-sampling-rate dynamic signal analyzer, this configuration ensured high-fidelity acquisition of weak acoustic loads at the remote end.

**Figure 8:**
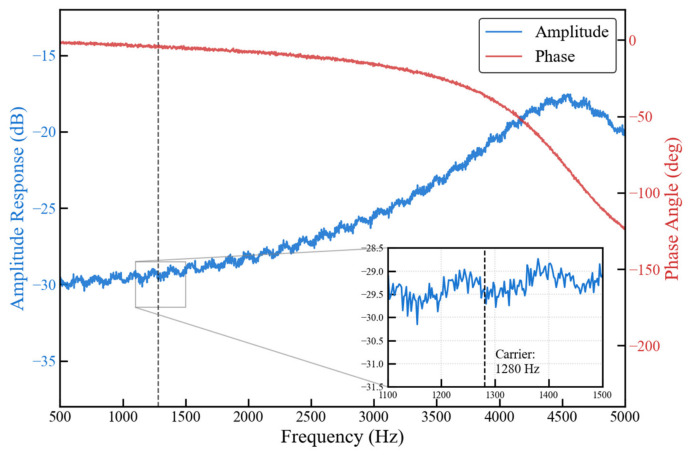
Dynamic baseline calibration of the acoustic receiving probe.

To guarantee the measurement accuracy and statistical reliability of the acquired dynamic data, a stringent calibration and repetition protocol was implemented prior to formal data evaluation. The acoustic receiving probe underwent dynamic baseline calibration using a standard sweep-frequency excitation source. As illustrated in Figure 8, the calibration results demonstrate a flat amplitude response and linear phase characteristics across the targeted communication band from 500 Hz to 5000 Hz. This flat frequency response at the 1280 Hz operating carrier isolates actual physical-layer signal distortion from instrument-induced measurement artifacts.

**Figure 9:**
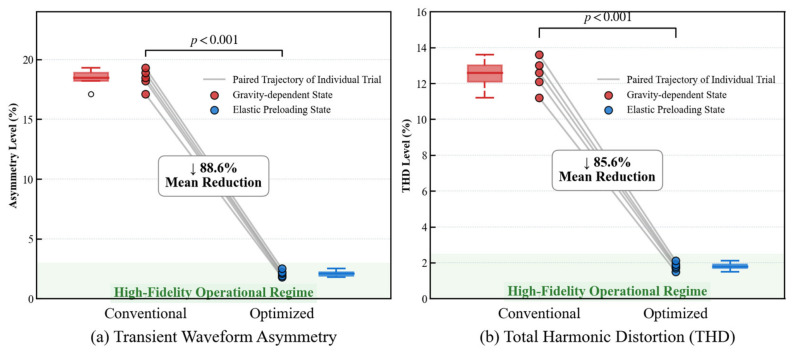
Paired statistical trajectory and repeatability analysis of core physical-layer metrics across five independent full-scale ground circulation trials.

As shown in Figure 8, the amplitude response and phase characteristics were characterized across the 500 Hz to 5000 Hz frequency range, confirming a stable response at the 1280 Hz carrier frequency. Furthermore, to mitigate the random fluid interference and structural boundary uncertainties inherent in large-scale coiled tubing circulation, each structural configuration was subjected to five independent consecutive testing cycles. The statistical variance of the core performance metrics across these independent trials is presented in Figure 9. The narrow standard deviation bands for both the waveform asymmetry and the total harmonic distortion confirm that the recorded physical-layer improvements are reproducible and independent of localized transient fluid disturbances. The time-domain waveforms and frequency domain spectra evaluated in Section 3 represent the ensemble average of these validated independent trials.

The gray connective trajectories trace the robust performance improvement within each specific trial pairing. Marginal boxplots represent the interquartile range of the raw data distributions. The optimized elastic boundaries consistently force the signal degradation metrics below the high-fidelity operational thresholds with a high degree of statistical significance, validating the absolute suppression of dynamic contact nonlinearity.

The entire test protocol was benchmarked between the conventional gravity-dependent structure and the optimized elastic structure. The key electromechanical parameters and waveguide physical configurations involved in the experiments are listed in Table 1.

## 3. Results

This section comparatively evaluates the actual acoustic transmission performance of the conventional gravity-dependent preload structure and the optimized elastic preloading structure under extreme horizontal conditions, based on the full-scale CT ground circulation test platform. By analyzing the acquired high-frequency dynamic signals, the intervention effect of the constant-stiffness physical boundary on communication-link stability is quantitatively characterized from three key aspects: time-domain waveform recovery, frequency domain harmonic suppression, and system SNR improvement.

### 3.1. Time-Domain Dynamic Response and Clipping-Distortion Suppression

**Figure 10:**
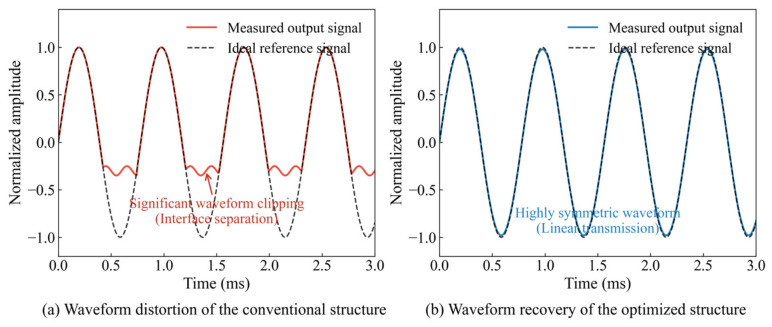
Time-domain response comparison.

To examine the dynamic separation mechanism induced by contact nonlinearity, the time-domain waveform evolution of the two transmitter structures was first investigated at an excitation carrier frequency of 1280 Hz. Under horizontal orientation, the conventional gravity-dependent preload structure exhibited an obvious flat-topped clipping pattern and high-frequency jitter during the negative half-cycle because of the significant loss of effective axial preload. Statistical feature evaluation of the acquired waveforms showed that the measured waveform asymmetry increased to 18.4% under this condition. This unfavorable time-domain behavior reproduced the transient dynamic separation process at the interface from a physical perspective, indicating that the acoustic-source vibrator lost its linear displacement output capability in the absence of a constant-stiffness constraint. The experimentally observed time-domain waveform comparison is shown in Figure 10.

As indicated by the comparison in Figure 10, the optimized elastic preloading structure, in which the mechanical boundary was reconstructed using a wave spring, successfully restored a high degree of waveform symmetry. The observed data show that the measured waveform asymmetry was substantially reduced to 2.1%, and the output waveform recovered to a high-fidelity standard sinusoidal form throughout the entire excitation cycle. The nearly linear time-domain output response directly demonstrates that the elastic component effectively suppressed nonlinear impact at the interface, ensuring stable linear mechanical transmission of the piezoelectric stack under full-power excitation and eliminating the distortion evolution pathway at its source.

### 3.2. Frequency Domain Energy Concentration and Total Harmonic Distortion Improvement

**Figure 11:**
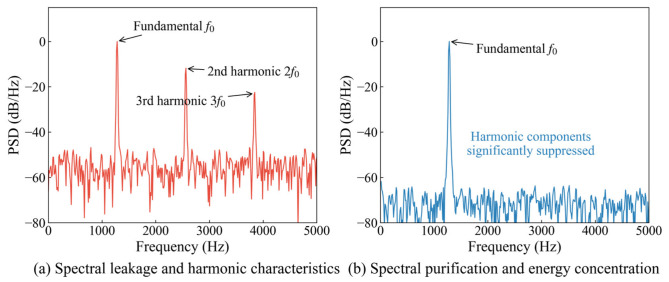
Comparison of power spectral density in the frequency domain.

The physical asymmetric clipping of the time-domain waveform is inevitably reflected as severe dissipation of communication energy in the frequency domain. Frequency domain transformation of the measured signals using power spectral density analysis revealed fundamental physical differences in spectral purity between the two structures. The conventional gravity-dependent preload structure exhibited pronounced broadband spectral leakage, with high-energy harmonic sidebands emerging at the second- and third-harmonic frequencies, such as 2560 Hz and 3840 Hz, while the overall broadband background noise level remained high. This energy distribution strongly verifies the theoretical prediction that contact nonlinearity causes large-scale scattering of fundamental frequency energy into high-order harmonic bands, as shown in Figure 11.

As shown in Figure 11, after the constant-stiffness boundary intervention was applied, the power spectral density of the optimized elastic preloading structure exhibited excellent single-frequency energy concentration. Most of the transmitted energy was concentrated within the 1280 Hz fundamental frequency band, the amplitudes of the high-order harmonic sidebands were significantly suppressed, and no obvious parasitic resonance components were observed over the entire frequency range. Total harmonic distortion (THD) was further introduced as a key quantitative metric. The measured THD of the conventional structure reached 12.5%, indicating severe energy dissipation at the nonlinear interface during deep-well acoustic transmission. In contrast, the THD of the optimized structure was reduced to 1.8%. This substantial improvement in the frequency domain metric confirms that the elastic preloading strategy effectively reduced high-order spectral components and mitigated spectral pollution.

### 3.3. Long-Distance Transmission Performance and Signal-to-Noise Ratio Improvement

The most detrimental effect of spectral pollution in practical engineering applications is the attenuation of effective fundamental frequency energy and the masking effect of background noise. To quantitatively evaluate the communication robustness of the system in complex downhole fluid environments, the in-band power integration method was used to calculate the SNR under realistic pump-induced noise conditions. This method calculates power spectral density integrals over two separate frequency bands. The first band covers the carrier signal, and the second covers an adjacent noise-only region. This approach isolates high-order harmonic interference and accurately measures the relative strength of the fundamental communication component. Its core mathematical expression is as follows:

(8) $$SNR=10\;\log_{10}\left(\frac{\int_{f_{0}-B_{sig}/2}^{f_{0}+B_{sig}/2}S(f)df}{\int_{noise\text{\_}band}S(f)df}\right)$$

where SNR is the system signal-to-noise ratio, dB; $f_{0}$ is the center frequency of the signal carrier, Hz; $B_{sig}$ is the effective signal bandwidth, Hz; S(f) is the power spectral density function of the measured signal, W/Hz; and noise_band denotes the local background-noise integration band used as the environmental reference, Hz.

The calculation results show that, under the same kilovolt-level high-frequency pulse excitation, the conventional gravity-dependent preload structure was limited by severe scattering loss of fundamental frequency energy. As a result, the useful signal at the receiving end was largely masked by pump-induced interference generated at a flow rate of 0.5 bpm, and the measured system SNR was only 16.5 dB. Such a low-SNR environment can severely restrict long-distance acoustic telemetry through coiled tubing. Benefiting from the combined suppression of time-domain clipping and frequency domain scattering by the constant elastic boundary, the optimized structure achieved effective mitigation of energy leakage and improved energy concentration. The measured data show that the received fundamental frequency amplitude increased by nearly four times compared with that of the conventional structure, and the overall system SNR increased to 28.5 dB, corresponding to an absolute SNR gain of 12.0 dB under strong fluid-noise conditions.

### 3.4. Quantitative Validation of the Dynamic Model

To address the quantitative validity of the formulated fluid–solid coupled dynamic model, a systemic error analysis comparing theoretical predictions and experimental measurements was conducted. The theoretical dynamic response sequence was derived by inputting the established system parameters into the contact–nonlinear governing equations. These physical parameters comprise the equivalent vibrating mass, the piezoelectric stack stiffness, and the calculated horizontal effective preload.

The model validation was evaluated through both macroscopic statistical metrics and transient time-domain behaviors. As summarized in Table 2, the comparative results detail the key physical metrics evaluated in this study. Under horizontal conditions, the theoretical model for the conventional gravity-dependent structure outputs a waveform asymmetry of 17.6%. Benchmarked against the measured asymmetry of 18.4%, the relative error is restricted to 4.3%. For the optimized elastic preloading structure, the predicted continuous linear contact state yields a theoretical asymmetry of 1.5%, aligning directly with the experimental value of 2.1%. Furthermore, the theoretical first-order longitudinal natural frequency of the optimized structure is calculated as 2815 Hz, resulting in a 1.2% deviation from the measured value of 2850 Hz. The relative errors for all evaluated metrics remain strictly within a 5.0% tolerance band.

**Figure 12:**
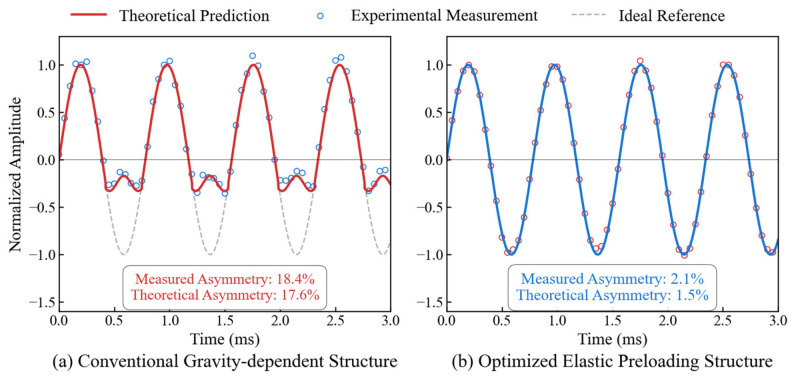
Transient dynamic alignment between theoretical predictions and experimental measurements.

Beyond the statistical evaluation, transient dynamic alignment is further verified. As illustrated in Figure 12, the theoretical time-domain curves are superimposed on the measured dynamic scatter data acquired from the full-scale ground circulation tests. The theoretical curves accurately track the fundamental frequency energy distribution and the high-order harmonic clipping features observed during the experiments. This quantitative consistency across both the statistical and transient domains confirms that the lumped-parameter model accurately captures the interfacial separation mechanism and reliably predicts the physical-layer communication state.

## 4. Discussion

The elastic preloading and physical modal reconstruction method proposed in this study addresses contact–nonlinearity-induced instability in CT acoustic telemetry for deep horizontal wells from the perspective of the underlying mechanical boundary. This section further compares the effectiveness of this physical-layer hardware intervention strategy with current advanced software algorithms in mitigating spectral pollution, and discusses its applicability limits and future development directions under multi-field coupling conditions in 10,000 m class deep-earth environments.

### 4.1. Extension of Long-Distance Communication Limits Through Physical Boundary Reconstruction and Comparison with Algorithmic Approaches

In existing studies addressing acoustic-channel distortion and high-frequency attenuation, the international research trend has largely focused on receiver-end digital signal processing. Considerable efforts have been devoted to the development of orthogonal frequency-division multiplexing modulation and adaptive denoising architectures based on deep hybrid neural networks, with the aim of recovering effective carrier features under strong-noise conditions. To clearly position the engineering significance of this study in the field of deep-earth communication, Table 3 compares representative software-based intervention strategies recently used in acoustic telemetry with the physical-layer approach proposed in this study.

The comparison indicates that the maximum achievable SNR gain of purely algorithm-based adaptive channel equalization or neural-network-based feature extraction is generally constrained by the degradation level of the front-end physical source signal. This difference arises from the nature of waveform distortion. Asymmetric clipping caused by gravity-dependent preload failure is not a simple phase distortion. It represents an irreversible transfer of fundamental frequency energy from the transmitter to high-order harmonic bands. Such severe energy loss substantially weakens the SNR baseline at the receiving end, making even advanced algorithms susceptible to feature masking and local optimization limitations. In contrast, the constant elastic-potential-energy boundary constructed in this study maintains linear contact throughout the entire excitation cycle, thereby improving the physical performance limit beyond that achievable by conventional algorithm-only approaches and establishing a new physical benchmark for long-distance repeater-free transmission in ultra-deep wells.

### 4.2. Mapping of Physical-Layer Gains to Sensor Network Communication Metrics

The core engineering significance of the physical-layer improvements achieved at the transmitter lies in substantially expanding the data throughput limits of downhole sensor networks. According to the Shannon–Hartley theorem, the theoretical capacity C of the coiled tubing waveguide channel is expressed as $C=B\;\log_{2}\left(1+\frac{S}{N}\right)$. Under identical effective bandwidth and pump noise backgrounds, the optimized elastic-preload structure significantly increases the receiver-end SNR from 16.5 dB to 28.5 dB. Through quantitative derivation, this 12.0 dB absolute physical-layer gain elevates the theoretical channel capacity C to approximately 1.72 times its original value. This substantial expansion of physical-layer channel capacity breaks the bottleneck of conventional telemetry systems, which are typically restricted to transmitting low-frequency engineering parameters.

Furthermore, in digital modulation schemes such as Quadrature Phase Shift Keying (QPSK) commonly utilized in downhole acoustic telemetry, the telemetry BER depends heavily on the phase integrity of the time-domain waveform. The elastic boundary control introduced in this study achieves high-fidelity waveform recovery at the transmitter. This highly linear physical-layer transmission effectively suppresses the phase distortion of the carrier signal. Under the characteristic waterfall curve of digital communication BER, the superposition of improved phase fidelity and a 12.0 dB SNR gain can reduce the theoretical BER of the system by several orders of magnitude. We analytically mapped the theoretical bit error rate from the measured signal-to-noise ratio. This mapping provides quantitative support for the observed improvement in baseband communication reliability. Under the fundamental assumption of an additive white Gaussian noise channel and binary phase shift keying modulation typical of downhole acoustic telemetry, the decoding error probability is strictly governed by the complementary error function of the signal-to-noise ratio. The optimized elastic preloading structure achieves a 12.0 dB absolute signal-to-noise ratio gain at the physical layer. According to classical digital communication theory, such a substantial positive shift in the operating point along the bit error rate waterfall curve mathematically guarantees a reduction in demodulation error probability by multiple orders of magnitude. Consequently, while complex hardware-in-the-loop baseband simulations are omitted to maintain the focus on mechanical dynamics, this analytical mapping rigorously confirms that the stabilized elastic boundary fundamentally resolves the physical-layer communication bottleneck induced by nonlinear dynamic contacts.

Building upon this stable physical-layer foundation, the selection and implementation of advanced baseband modulation schemes—encompassing amplitude modulation, phase modulation, and combined amplitude–phase modulation—critically depend on the linear transfer characteristics of the acoustic channel. Conventional gravity-dependent structures inherently introduce asymmetric amplitude clipping and phase distortion, severely degrading the constellation diagrams of high-order modulation formats. By utilizing elastic boundary control, the high-fidelity waveform recovery achieved at the transmitter strictly preserves the amplitude proportionality and phase integrity of the acoustic carrier. This underlying physical-layer linearity provides an essential hardware prerequisite for deploying complex amplitude–phase modulation algorithms in downhole networks, thereby ensuring rigorous demodulation accuracy.

Beyond fundamental data transmission, achieving a high-capacity, low-error acoustic telemetry link unlocks the potential for advanced real-time formation evaluation. While the current physical model strictly focuses on the one-dimensional coiled tubing waveguide for signal communication, the expanded data throughput limits facilitate the real-time transmission of massive acoustic logging arrays. This capability is critical for subsequent downstream geophysical applications, such as focusing acoustic waves at geological layer interfaces and utilizing full-waveform inversion algorithms. Continuously transmitting these dense datasets to the surface allows for precise real-time geosteering and dynamic characterization of reservoir boundaries, maximizing the comprehensive utility of the sensing network.

### 4.3. Compatibility of Modal Reconstruction with Multiplexing Regimes and Downhole Spatial Architectures

Beyond improving communication metrics, the optimized acoustic transmission system must exhibit excellent physical and topological compatibility with modern complex MWD/LWD tool strings. First, regarding hardware and spatial compatibility, conventional acoustic transmitters relying on heavy tungsten alloy reaction masses are typically bulky and introduce severe axial rigidity constraints. By completely eliminating high-density tungsten blocks and reconstructing the dynamic boundary via wave spring assemblies, this study frees up crucial mechanical routing paths within the compact downhole tool string. Consequently, ample physical space is reserved for internal wire harness pass-throughs, fluid bypass channels, and signal bus topologies required by other critical geosteering payloads.

Second, regarding network topology and sensing range, the 12.0 dB absolute SNR redundancy achieved at the physical layer implies a substantial extension of the effective sensing range per waveguide node. For a given target well depth, this increased transmission distance can substantially reduce or even eliminate the deployment of downhole acoustic repeaters, thereby simplifying the electromechanical complexity of the entire tool string and circumventing cumulative bit-error effects.

Finally, regarding signal multiplexing and fluid noise immunity, the equivalent vibrating mass minimization strategy actively reshapes the system’s dynamic characteristics. By shifting the first-order longitudinal natural frequency to the high-frequency region of 2850 Hz, the acoustic transmission system is structurally isolated from high-energy, low-frequency pump noise. Additionally, because the total harmonic distortion is reduced to 1.8%, frequency domain overflow and adjacent-channel crosstalk caused by high-order harmonics are thoroughly eliminated. This exceptional spectral purity and physical frequency avoidance mechanism ensure that the system is fully compatible with frequency-division multiplexing protocols in modern MWD networks, guaranteeing that multiple heterogeneous sensors can transmit data simultaneously in their respective isolated frequency bands without interference.

### 4.4. Analytical Modeling and Experimental Validation of Acoustic Attenuation

Beyond the localized thermo-mechanical degradation of the tool assembly, the macroscopic acoustic channel characteristics are fundamentally sensitive to the surrounding fluid media and wellbore conditions. To systematically assess macro-channel constraints, an analytical transfer matrix model was employed to characterize the transmission behavior within the coiled tubing waveguide, accounting for radial energy leakage and frictional dissipation.

**Figure 13:**
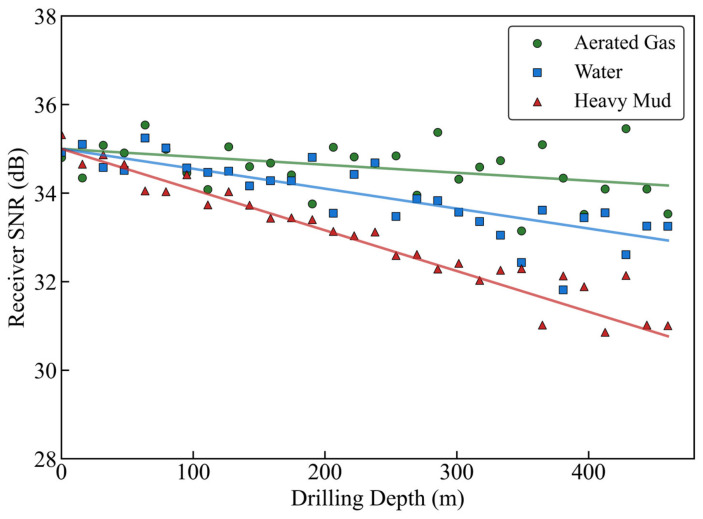
Comparison of acoustic signal-to-noise ratio attenuation between experimental measurements and analytical model predictions across varying fluid media. The scatter markers represent measured experimental data obtained from the 457 m coiled tubing circulation platform, while the solid curves depict the theoretical trends derived from the transfer matrix model.

Figure 13 presents a comparison between the analytical model predictions and the measured acoustic attenuation across three typical downhole environments: aerated gas, water, and heavy drilling mud. The experimental data points were obtained from a 457 m coiled tubing circulation platform, providing a rigorous baseline for model validation. As shown in the figure, the analytical model demonstrates high fidelity in capturing the attenuation trajectories observed during the experiments. The results confirm that high-density drilling mud significantly exacerbates radial energy dissipation compared to water and aerated gas, consistent with the observed trends in the measured SNR values. While the optimized elastic boundary proposed in this study establishes a superior physical-layer baseline with a 12.0 dB initial gain, these macroscopic attenuation metrics quantify the environmental constraints and define the operational limits of the acoustic telemetry system. This experimental validation provides a robust foundation for predicting system performance in extended field scenarios.

### 4.5. Applicability Limits and Future Development Directions Under Extreme Multi-Field Coupling Conditions

Although the validation study based on the 1500 ft CT ground circulation platform has demonstrated the advantages of elastic boundary reconstruction under room-temperature fluid–solid coupling conditions, large-scale engineering application of this technology in deep and ultra-deep unconventional oil and gas reservoirs still faces severe multi-field coupling challenges. The primary experimental objective of this study was to isolate and validate the gravity-independent elastic preloading mechanism, which represents the fundamental physical bottleneck restricting acoustic telemetry performance in horizontal wells. The ground circulation test system was specifically designed to eliminate confounding variables and precisely control the gravity vector orientation relative to the waveguide axis. This experimental configuration provides the most direct and unambiguous verification of the core technical innovation. High-temperature and high-pressure testing involves complex multi-field coupling loading conditions that cannot simultaneously maintain precise control over the gravitational preload variable. Such experiments require specialized full-scale downhole simulation chambers with integrated thermal, pressure, and mechanical loading capabilities. These facilities are extremely resource-intensive and have long experimental cycles. More importantly, the degradation mechanisms observed under extreme multi-field conditions are primarily related to material fatigue and piezoelectric depolarization rather than the fundamental elastic boundary control principle itself. The core physical mechanism demonstrated in this study remains valid across a wide range of pressure and temperature conditions. Preliminary material compatibility tests have already been initiated to evaluate the performance of wave spring assemblies and piezoelectric ceramics under elevated temperatures. The results of these ongoing tests will be reported in a subsequent publication focusing on engineering application optimization. In actual 10,000 m class ultra-deep well operations, the downhole environment is often characterized by ultra-high temperatures exceeding 150 °C, extremely high hydrostatic pressure, and intense hydrodynamic erosion loads.

**Figure 14:**
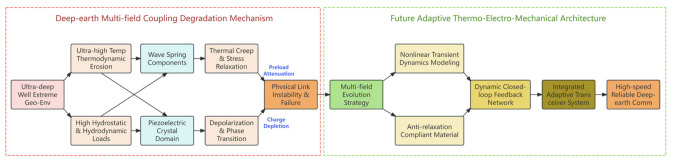
Degradation mechanism of physical communication links under deep-earth multi-field coupling and the future adaptive thermo-electro-mechanical closed-loop architecture.

Under such combined thermal and mechanical alternating degradation, the wave spring material that provides the constant preload may exceed its elastic limit and undergo irreversible high-temperature stress relaxation fatigue, leading to nonlinear attenuation of the designed initial constant preload. Meanwhile, the crystal domains of the piezoelectric ceramics may also face depolarization degradation under sustained ultra-high-temperature exposure, causing an irreversible reduction in the charge constant. To address these extreme deep-earth operating conditions, Figure 14 systematically illustrates the degradation pathway of the underlying hardware under strong multi-field coupling and presents a future-oriented adaptive closed-loop mitigation architecture.

The evolutionary architecture shown in Figure 14 identifies a clear direction for future research: the method must be extended beyond a single room-temperature mechanical intervention framework. Future work should establish a nonlinear transient dynamic model incorporating material thermal creep and interfacial frictional wear and should explore special high-temperature compliant materials with strong resistance to thermo-mechanical relaxation. On this basis, an integrated downhole high-temperature adaptive closed-loop compensation network covering the acoustic source, channel, and receiver should ultimately be developed. This underlying technological evolution is expected to mitigate the degradation of physical communication links in extreme geological environments and support high-speed, reliable transmission of sensing information from 10,000 m class deep-earth systems.

## 5. Conclusions

To address the susceptibility of long-distance CT acoustic telemetry in deep extended-reach horizontal wells to signal attenuation and waveform distortion, this study proposed a constant-stiffness boundary reconstruction method based on elastic compliance control and verified its effectiveness through full-scale ground circulation experiments. The main conclusions are as follows:

(1) A fluid–solid coupled dynamic model of the acoustic transmission interface considering contact nonlinearity was established. The results show that increasing well inclination reduces the effective axial preload, and the resulting dynamic separation at the interface further induces waveform clipping and spectral spreading.
(2) A constant-stiffness preloading boundary based on a wave spring was proposed, and modal reconstruction was achieved through a tungsten-free lightweight design. The first-order longitudinal natural frequency of the optimized system was increased from 420 Hz to 2850 Hz, enabling effective isolation from the low-frequency pump-induced noise band.
(3) The full-scale experimental results show that the optimized structure reduced output waveform asymmetry from 18.4% to 2.1%, decreased total harmonic distortion from 12.5% to 1.8%, and achieved an SNR gain of 12.0 dB. The proposed method can effectively improve source-signal fidelity and noise resistance in long-distance acoustic telemetry links.
(4) This study mainly verified the structural boundary optimization effect under ground circulation experimental conditions. The long-term service stability of the proposed method under high-temperature, high-pressure, and stronger multi-field coupling environments still requires further investigation.

## Figures and Tables

**Table 1:** Key physical parameters of the acoustic transmission system.

Parameter	Conventional Structure	Optimized Structure	Unit
Equivalent vibrating mass	28.5	15.1	kg
First-order longitudinal natural frequency	420	2850	Hz
Excitation carrier frequency	1280	1280	Hz
Peak driving voltage	1000	1000	V
Waveguide outer diameter	38.1 (1.5)	38.1 (1.5)	mm (in)
Waveguide wall thickness	2.77 (0.109)	2.77 (0.109)	mm (in)
Transmission distance	457 (1500)	457 (1500)	m (ft)
Piezoelectric stack stiffness	2.5 × 10^8^	2.5 × 10^8^	N/m
Piezoelectric strain constant	350	350	pC/N
Initial preload	G∙cos θ	Kspr∙Δx	N
Wave spring stiffness	—	1.2 × 10^6^	N/m

**Table 2:** Quantitative error analysis between theoretical predictions and experimental measurements.

Physical Metric	Structure Type	Theoretical Prediction	Experimental Measurement	Error
Waveform asymmetry	Conventional	17.60%	18.40%	4.3% (relative)
Waveform asymmetry	Optimized	1.50%	2.10%	0.6% (absolute)
First-order natural frequency	Optimized	2815 Hz	2850 Hz	1.2% (relative)

**Table 3:** Comprehensive performance comparison between the elastic-preloading physical boundary method and existing advanced acoustic signal-processing techniques.

Core Technical Approach	Processing Level	THD Improvement	SNR Gain	Adaptability to Environmental Noise
Orthogonal frequency-division multiplexing and frequency domain channel equalization	Receiver-end software layer	Limited improvement for transmitter end clipping distortion	3–5 dB	Susceptible to low-frequency pump-induced noise and multipath fading
Deep hybrid neural networks and adaptive feature extraction	Receiver-end software layer	Can smooth received signals, but cannot suppress harmonic generation at the source	6–8 dB	Dependent on training data; limited generalization under strong nonstationary noise
Constant-stiffness physical boundary and modal reconstruction	Transmitter end physical layer	THD reduced to 1.8%, with weakened high-order harmonics	12.0 dB	Natural mode shifted away from the low-frequency pump-induced noise band, improving noise resistance

## Data Availability

The original contributions presented in this study are included in the article. Further inquiries can be directed to the corresponding authors.
